# Cellular Depletion of BRD8 Causes p53-Dependent Apoptosis and Induces a DNA Damage Response in Non-Stressed Cells

**DOI:** 10.1038/s41598-018-32323-3

**Published:** 2018-09-20

**Authors:** Anahita Lashgari, Myriam Fauteux, Alexandre Maréchal, Luc Gaudreau

**Affiliations:** Département de biologie, Université de Sherbrooke, 2500 Boulevard de l’Université, Sherbrooke, QC J1K 2R1 Canada

## Abstract

Regulation of the chromatin state is crucial for biological processes such as the regulation of transcription, DNA replication, and DNA damage repair. Here we show that knockdown of the BRD8 bromodomain protein – a subunit of the p400/Tip60 complex - leads to *p21* induction, and concomitant cell cycle arrest in G1/S. We further demonstrate that the p53 transcriptional pathway is activated in BRD8-depleted cells, and this accounts for upregulation of not only *p21* but also of pro-apoptotic genes, leading to subsequent apoptosis. Importantly, the DNA damage response (DDR) is induced upon BRD8 depletion, and DNA damage foci are detectable in BRD8-depleted cells under normal growth conditions. Consistently with an activated DDR, we find that in BRD8-depleted cells, the ATM-CHK2 DDR pathway is turned on but, CHK1 proteins levels are severely reduced and replication stress is detectable as enhanced replication protein A (RPA32) phosphorylation levels. Notably, acetylation of histone H4 at K16 (H4K16ac) is reduced in BRD8-depleted cells, suggesting that BRD8 may have a role in the recruitment and/or stabilization of the p400/Tip60 complex within chromatin, thereby facilitating DNA repair. Taken together, our results suggest that BRD8 is involved not only in p53-dependent gene suppression, but also in the maintenance of genome stability.

## Introduction

Dynamic changes in chromatin structure are an inevitable necessity in many cellular processes such as gene transcription, DNA replication, DNA repair and recombination. Chromatin dynamics can be modulated through different mechanisms including post-translational modification of histone tails, physical displacement of nucleosomes by ATP-dependent chromatin remodelers, and exchange of canonical histones by histone variants^1,2^. Histone post-transcriptional modifications alter the structure of chromatin and act as docking sites for regulatory proteins that specifically recognize these modifications to recruit or stabilize factors involved in chromatin-associated processes such as nucleosome remodeling. Amongst histone modifications, lysine acetylation is a very dynamic modification which directs structural changes in chromatin as well as modulates gene transcription^3,4^. Emerging evidence suggests that histone acetylation plays an important role in DNA repair and replication, but the precise mechanism remains to be elucidated^5^. Lysine acetylation on histone tails creates docking sites for bromodomain (BRD) -containing proteins^6^. BRDs are an important family of readers of lysine acetylation and they can recognize acetylated-lysine residues on proteins including histone tails^6,7^. Dysfunction of BRD-containing proteins has been linked to pathological conditions, including cancer, inflammation and viral replication^7^. Even though recent studies have highlighted the roles of BRDs in various biological processes and their association with disease, the functions of many human BRD proteins, such as BRD8, are not well characterized.

The human BRD8 gene is expressed predominantly as two main isoforms. Isoform 2 is larger (135.4 kDa) than isoform 1 (102.8 kDa). Both isoforms are subunits of the p400/Tip60 chromatin remodeler/Histone Acetyl Transferase (HAT) complex comprising at least 16 subunits, including p400 and Tip60^8,9^. p400 is a SWR1- class ATP-dependent remodeling protein that deposits the histone variant H2A.Z into specific regions of chromatin. Tip60 is a histone acetyl transferase that acetylates histone H4, H2A and H2A.Z, as well as non-histone proteins^10^. P400/Tip60 remodeling activity is crucial for the regulation of gene expression, cell cycle progression, and DNA repair (reviewed in^4^).

BRD8 appears to be involved in the regulation of cancer cell proliferation and the response to chemotherapeutic compounds, which destabilize the cytoskeleton or impede proteasomal function^11^. The expression level of BRD8 is elevated several-fold in metastatic colorectal cancer cells compared to non-aggressive colorectal adenocarcinoma or slowly proliferating colorectal tumor cells^11^. BRD8 overexpression confers improved proliferation and is correlated with invasiveness and aggressiveness of cancerous cells and their resistance to nocodazole, taxol and MG132^11^. Contrastingly, BRD8 knockdown induces cell death or growth delay in colorectal and prostate cancer cells, and cells surviving BRD8 knockdown are more sensitive to microtubule-depolymerizing agents^11–13^. However, the mechanisms through which BRD8 controls cell proliferation, apoptosis and drug resistance in tumor cells are still poorly understood but an intriguing possibility is that this component of the p400/Tip60 complex may participate in genome maintenance.

Repair of damaged DNA requires the remodeling of local chromatin structure which provides access to the site of DNA damage for the repair machinery^14,15^. In recent years, chromatin remodeling complexes, histone modifications and dynamic changes in nucleosome organization have been recognized as active players in the process of efficient DNA damage repair^15^. The p400/Tip60 remodeling complex plays a key role in repair of DNA double-stranded breaks (DSBs) and maintenance of genome stability^10^. Loss of functional p400/Tip60 leads to defective DNA double-stranded breaks DSBs repair and increased sensitivity to DNA damaging agents^16–18^. Components of the p400/Tip60 complex are actively recruited to DSBs to acetylate H4, H2A and H2AX thereby facilitating chromatin opening^19–21^. In addition, H2A.Z is transiently exchanged into nucleosomes at DSBs by the p400 remodeling complex and shifts the chromatin to an open conformation which is required for acetylation and ubiquitination of histones and for loading of the DNA repair proteins^22–24^. However, little is known about the function of BRD8 as a subunit of the p400/Tip60 complex in the context of damaged DNA.

In the present study, we have investigated the molecular mechanisms underlying growth defects and cell death in BRD8-depleted human colorectal cancer cells (HCT116). Here we report that cellular depletion of BRD8 by siRNA-induced cellular depletion increases the expression of *p21*, which leads to a cell cycle arrest in G1/S and subsequent p53-dependent apoptosis. The growth deficiency induced by BRD8 knockdown is also observed in p53−/− and p21−/− cells. In p53−/− and p21−/− cells, BRD8-depleted cells accumulate in the G2 phase of the cell cycle suggesting the existence of un-repaired DNA damage. Indeed, we provide evidence that DNA damage accumulates following BRD8 knockdown. This DNA damage results in the activation of the ATM-CHK2 DDR pathway. Interestingly, BRD8 knockdown also results in the reduction of CHK1 protein levels and induction of replicative stress. Taken together, our results suggest that BRD8 is required for genome maintenance and prevents DNA damage accumulation in non-stressed cells.

## Results

### BRD8 knockdown induces cell cycle arrest and cell death through induction of p53-dependent apoptosis in HCT116 cells

**Figure 1:**
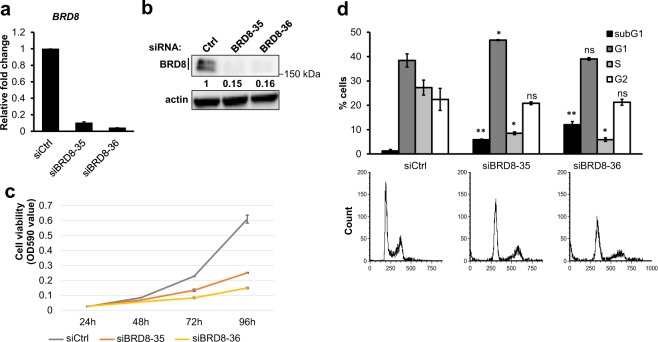
BRD8 knockdown induces cell cycle arrest and cell death. BRD8 depleted in HCT116 cells with two siRNAs targeting BRD8 (siBRD8-35 and siBRD8-36) or control siRNA (Ctrl) for 48 h. (**a**) Expression levels of *BRD8* in HCT116 p53+/+ cells before and after knockdown using two independent siRNAs. (**b**) Immunoblot showing BRD8 expression in BRD8 knockdown cells. Numbers represent densitometric analysis normalized to actin. (**c**) Crystal violet staining viability assay of HCT116 p53+/+ cells at 24, 48, 72 and 96 h post transfection with siRNAs targeting BRD8 (siBRD8-35 and siBRD8-36) or control siRNA (Ctrl). Three independent experiments were performed in technical duplicates; the mean ± SD is shown. (**d**) Cell cycle distributions of HCT116 p53+/+ following BRD8 knockdown for 72 h and analyzed by PI staining FACS. The mean of 3 independent experiments ± SD is shown. An ANOVA test followed up with a Dunnett analysis was used to compare each mean to its relative control. *P ≤ 0.05; **P ≤ 0.01; ns = non-significant compared to relative control.

BRD8 was previously shown to be over-expressed in metastatic and highly proliferating colorectal cancer cell lines. It has also been proposed that BRD8 expression is associated with tumor progression towards advanced stages by providing a growth advantage^11^. To investigate a possible function of elevated BRD8 expression in the proliferation of cancer cells, we depleted BRD8 in HCT116 cells. Two siRNAs targeting different regions of the BRD8 mRNA were used. Transfection of HCT116 cells with both siRNA constructs significantly reduced BRD8 mRNA transcription after 48 hours of transfection as measured by RT-qPCR to levels equivalent to 4–10% of control cells (Fig. 1a). The protein levels of both BRD8 isoforms also decreased strongly to ~15% of control cells after transfection with both siRNA constructs (Fig. 1b).

To characterize the effects of BRD8 depletion on cellular fitness, we first monitored cell viability by crystal violet staining at 24, 48, 72, and 96 hours after transfection. Consistent with previous results^11^, depletion of BRD8 in HCT116 p53+/+ cells significantly reduced the number of viable cells compared to the control siRNA after 72 h of siRNA transfection (Fig. 1c). To investigate the mechanism(s) underlying the decrease in viability following BRD8 knockdown, we analyzed the cell cycle distribution of transfected cells. Cell cycle analysis showed that transfection of HCT116 cells with BRD8 siRNA significantly reduced the cell population in S-phase (S-phase: 27.25 ± 3.07% in control transfection to 8.50 ± 0.57% and 5.89 ± 0.77% in BRD8 siRNA transfection) (Fig. 1d). In agreement with a previous study^11^, knockdown of BRD8 also induced an increase of the cell population in sub-G1 (sub-G1: 1.28 ± 0.56% in control transfection to 5.88 ± 0.23% and 12.01 ± 1.31% in siBRD8), suggesting an increase in DNA fragmentation and cell death compared to control (Fig. 1d). Thus, we reasoned that the observed decrease in viability might be caused by G1/S arrest as well as induction of cell death in HCT116 p53+/+ cells.

**Figure 2:**
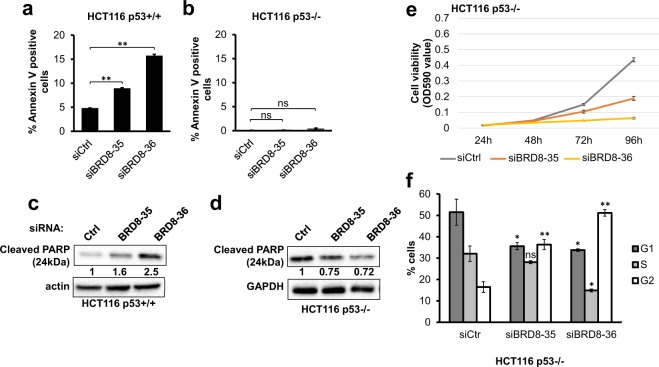
BRD8 knockdown induces p53-dependent apoptosis in p53+/+ cells and G2 arrest in p53−/− cells. Apoptosis in HCT116 p53+/+ (**a**) and HCT116 p53−/− (**b**) cells were quantified by FACS analysis of Annexin V and PI double staining 72 h post transfection with two independent siRNAs targeting BRD8 (siBRD8-35 and siBRD8-36) (or control siRNA (Ctrl)). Immunoblot showing the cleaved PARP in HCT116 p53+/+ (**c**) and HCT116 p53−/− (**d**) cells in BRD8 depleted cells. Numbers represent densitometric analysis normalized to actin. (**e**) Crystal violet staining assay of HCT116 p53−/− cells at 24, 48, 72 and 96 h post transfection with siRNAs targeting BRD8 (siBRD8-35 and siBRD8-36) or control siRNA (CT), the cells were fixed and stained with crystal violet. Crystal violet stain was dissolved and optical absorbance was measured at 590 nm (OD590). Three independent experiments were performed; the mean ± standard deviation is shown. (**f**) Cell cycle distributions of HCT116 p53−/− following BRD8 knockdown for 72 h and analyzed using flow cytometry. Data are the mean ± SD from three independent experiments. An ANOVA test followed up with a Dunnett analysis was used to compare each mean to its relative control. *P ≤ 0.05; **P ≤ 0.01; ns = non-significant compared to relative control.

Given that the knockdown of BRD8 leads to decreased cell survival and increased sub-G1 populations, we wanted to see if this correlated with the induction of apoptosis. For this purpose, Annexin V-FITC/propidium iodide (PI) staining of BRD8-depleted HCT116 cells was performed. The results indicate that BRD8 knockdown reproducibly caused a significant increase in the population of Annexin V positive PI negative cells (early apoptotic cells) in HCT116 p53+/+ cells (Fig. 2a). We also assayed PARP cleavage, which is an indicator of caspase activation and apoptosis^25,26^. Immunoblot analysis showed that, in BRD8-depleted HCT116 p53+/+ cells, PARP protein cleavage was enhanced (Fig. 2c).

Since apoptosis may occur in either a p53-dependent or -independent manner, we tested whether the observed induced apoptosis was p53-dependent. For this purpose, we depleted BRD8 in isogenic HCT116 p53−/− cells. Knockdown of BRD8 in HCT116 p53−/− cells did not significantly induce apoptosis (Fig. 2b), nor was there any increase in PARP cleavage detected in those cells following BRD8 knockdown (Fig. 2d). Similar results were obtained in both HCT116 p53+/+ and p53−/− cells, respectively when using shRNA-mediated BRD8 knockdown instead of siRNA-mediated knowckdown (Fig. S1). These data indicate that BRD8 depletion induces p53-dependent apoptosis in HCT116 cells.

Next, we monitored cell viability following BRD8 knockdown in p53−/− cells. Despite the lack of apoptosis, similarly to what we observed in HCT116 p53+/+ cells, BRD8 knockdown significantly reduced the number of viable cells compared to control siRNA in HCT116 p53−/− cells (Fig. 2e). We further analyzed the effect of BRD8 knockdown on cell cycle distribution in HCT116 p53−/− cells. As shown in Fig. 2f BRD8 knockdown in p53−/− cells caused a G2 arrest. G2 cell populations significantly increased in both siBRD8-transfected cells when compared with that of control cells. (G2: 16.47 ± 2.48% in control transfection to 36.31 ± 2.34% and 51.18 ± 1.52% in siBRD8). Taken together our data suggests that in p53−/− cells like in p53+/+ cells, BRD8 may be involved in cell cycle progression.

**Figure 3:**
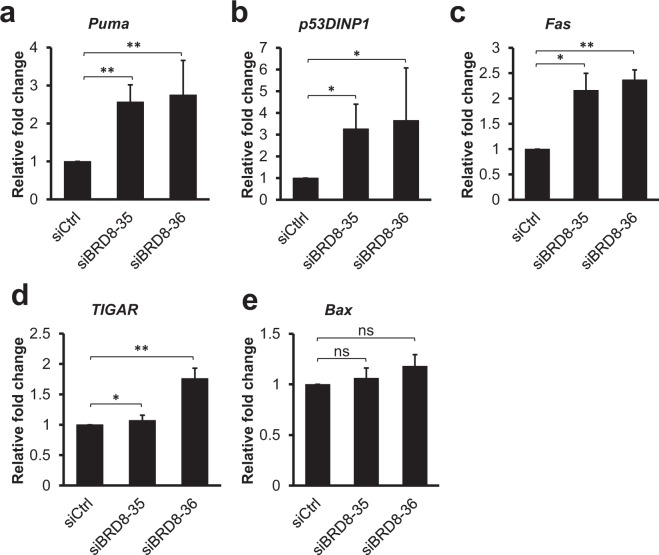
BRD8 knockdown induces pro-apoptotic p53 target genes in HCT116 cells. (**a**–**d**) RT-qPCR assay showing mRNA expression levels of *Puma*, *p53DINP1*, *Fas*, *TIGAR*, *Bax*. Data are the mean ± SD from three independent experiments. An ANOVA test followed up with a Dunnett analysis was used to compare each mean to its relative control. *P ≤ 0.05; **P ≤ 0.01; ns = non-significant compared to relative control.

In order to identify the molecular mechanism of apoptosis following BRD8 knockdown, we investigated the effect of BRD8 depletion on the transcription of p53 pro-apoptotic target genes. Among p53 target genes, *p21* and *Puma* (p53 upregulated mediator of apoptosis) are major mediators of p53 tumor suppressor effects, such as growth arrest and apoptosis. Puma, a pro-apoptotic BCL-2 family protein, was previously shown to be required for the induction of p53-dependent apoptosis, and it is directly induced by p53 in response to stress stimuli such as DNA damage^27^. As shown in Fig. 3a, siRNA-mediated knockdown of BRD8 significantly induced the transcription of *Puma*. Next, we assayed the expression of *p53DINP1* (p53-dependent damage-inducible nuclear protein 1), which is responsible for the induction of apoptosis in response to DSBs^28^. As shown in Fig. 3b, knockdown of BRD8 significantly induced the transcription of *p53DINP1*. In addition, p53 activates the extrinsic apoptotic pathway through the transcriptional induction of the trans-membrane proteins, such as cell surface receptors Fas, which is a member of the TNF-R family of receptors^29^. Knockdown of BRD8 significantly induced *Fas* transcription (Fig. 3c). In addition, *TIGAR* (*TP53*-induced glycolysis and apoptosis regulator) mRNA was also increased following depletion of BRD8 by our most potent BRD8-depleting siRNA 36 (Fig. 3d). We also measured the transcription of another p53-inducible pro-apoptotic gene, *Bax*^30,31^. Whereas transcription of *Puma*, *Fas* and *p53DINP1* are significantly induced and *TIGAR* mRNA was increased by our most potent BRD8-depleting siRNA 36, *Bax* was unaffected following depletion of BRD8 (Fig. 3e). The requirement for *Bax* in p53-mediated apoptosis is cell type-dependent. It has been shown that *Bax* is not essential for apoptosis in response to irradiation in mouse colonic epithelia cells^32^. Taken together, these results suggest that the induction of apoptosis by cellular depletion of BRD8 is the result of the transcriptional activation of pro-apoptotic p53 target genes.

### Cellular depletion of BRD8 induces p53-dependent *p21* transcription in HCT116 cells

**Figure 4:**
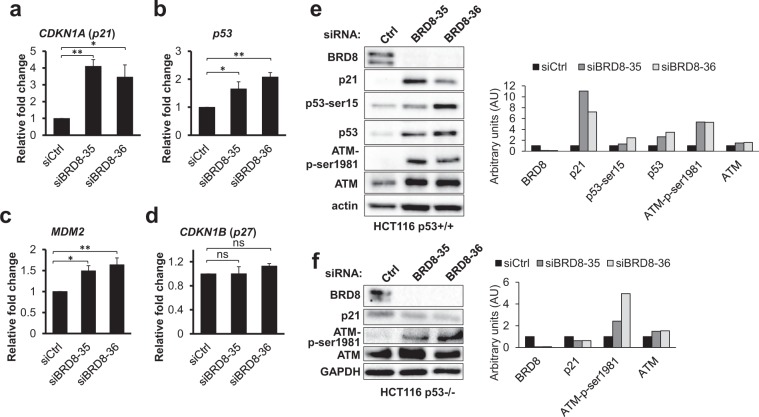
Knockdown of BRD8 activates p53 in HCT116 p53+/+ cells. (**a**–**d**) BRD8 was depleted in HCT116 cells with two siRNAs targeting BRD8 (siBRD8-35 and siBRD8-36) or control siRNA (Ctrl) for 48 h. Total RNA was extracted and RT-qPCR assays were performed. mRNA expression levels of *p21*, *p53*, *MDM2* and *p27* were monitored. The mean ± SD from three independent experiments are shown. Total cell extracts of HCT116 p53+/+ (**e**) and HCT116 p53−/− (**f**) were subjected to immunoblot assays using the indicated antibodies. The graphics represent densitometric analyses normalized to *actin* or *GAPDH*. An ANOVA test followed up with a Dunnett analysis was used to compare each mean to its relative control. *P ≤ 0.05; **P ≤ 0.01; ns = non-significant compared to relative control.

In order to provide insight into the molecular mechanisms involved in G1/S cell cycle arrest, we assayed whether the transcription of cell cycle regulating genes is affected by depletion of BRD8. Cell cycle progression is regulated by three protein families: Cyclins, cyclin-dependent kinases (CDKs), and CDK inhibitors (CDKIs). G1/S transition is triggered by the formation of cyclin D1-CDK4/6 complexes, which regulate the synthesis of DNA in preparation for cell division, while the CDKIs *CDKN1A* (*p21*) and *CDKN1B* (p27) have a negative regulatory role in this process^33,34^. In fact, cyclin-dependent kinase inhibitor *p21* is essential for G1/S cell cycle arrest^35^. Depletion of BRD8 significantly increased *p21* mRNA (Fig. 4a). We also examined p21 protein levels in BRD8-depleted cells by immunoblot. In agreement with our RT-qPCR data, p21 protein levels increased in BRD8-depleted cells compared to control cells (Fig. 4e). Another key component in the regulation of cell cycle progression is p53, and when activated by genotoxic stresses, p53 directly up-regulates the *p21* gene to inhibit cell cycle progression^34^. As expected from our apoptosis data, analysis of *p53* expression indicated that both mRNA (Fig. 4b) and protein levels (Fig. 4e) of *p53* are elevated in BRD8-depleted cells. To evaluate whether BRD8 depletion activates the p53 transcriptional pathway, we also examined the expression of *MDM2*, which is a canonical transcriptional target of p53 that provides negative feedback regulation of p53 activity^36^. As shown in Fig. 4c, knockdown of BRD8 induced the transcription of *MDM2* in response to elevated p53 expression. The regulation of *p21* gene transcription not only occurs through p53 but also in a p53-independent pathway^34^. We therefore asked whether BRD8 siRNA-mediated *p21* expression is exclusively p53-dependent. For this purpose, HCT116 p53−/− cells were used and knockdown of BRD8 did not increase the basal levels of *p21* protein in HCT116 p53−/− cells, indicating that the induction of *p21* is p53-dependent (Fig. 4f).

We also assayed the mRNA level of the other member of the *Cip/Kip* family of cyclin-dependent kinase inhibitors, p27 (*CDKN1B*), which controls cell cycle progression in G1, by RT-qPCR. As shown in Fig. 4d, *p27* mRNA levels were unchanged in BRD8 knockdown cells compared to control cells. Hence, it can be assumed that the G1/S arrest seen in the BRD8-depleted cells is due to increased *p21* expression level.

**Figure 5:**
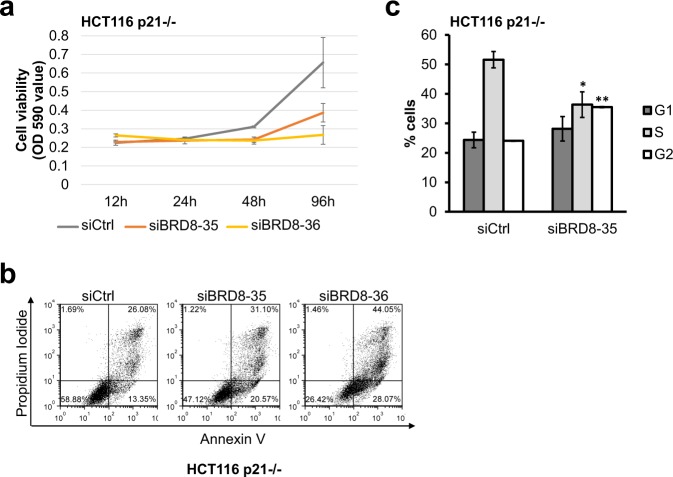
Lack of *p21* does not rescue proliferation arrest and apoptosis in BRD8-depleted cells. (**a**) Crystal violet staining assay of HCT116 p21−/− cells at 24, 48, 72 and 96 h post transfection with BRD8-targeting (siBRD8-35 and siBRD8-36) or control (Ctrl) siRNAs. Three independent experiments were performed; the mean ± SD is shown. (**b**) Apoptosis in HCT116 p21−/− cells were quantified by FACS analysis of Annexin V and PI double staining 72 h post transfection. (**c**) Cell cycle distributions of HCT116 p21−/− following BRD8 knockdown for 72 h and analyzed by PI staining FACS. The mean of three independent experiments ± SD is shown. Two-sample t-Test assuming unequal variances was used for statistical analysis. *P ≤ 0.05; **P ≤ 0.005 compared to control.

Previous studies from our laboratory have demonstrated that p400-dependent deposition of H2A.Z at the distal p53-binding site of *p21* promoter inhibits p53-dependent *p21* transcription and thereby prevents replicative senescence^37^. Previously, we have showed that knockdown of H2A.Z results in an increase in *p21* expression and cellular senescence^37^. Moreover, the decrease in the expression of p400 was shown to induce *p21* expression and cellular senescence through the p53/p21 pathway^37,38^. In addition, loss of MRG15, another component of the p400/Tip60 complex, was shown to limit neural stem/progenitor cell proliferation via increased expression of *p21*^39^. Thus, we investigated this possibility in BRD8-depleted HCT116 cells, since the bromodomain protein is a subunit of the p400/Tip60 complex^8^. As shown in Fig. S2, transcription levels of p400, Tip60 and MRG15 were not affected by cellular depletion of BRD8 (Fig. S2a–c). Despite the fact that H2A.Z transcription was decreased by BRD8 depletion, the protein levels of H2A.Z were not affected by the knockdown of BRD8, at least not during the course of our experiments (Fig. S2d,e). This suggests that the increase in *p21* expression in BRD8-depleted cells is specific and it not due to changes in expression and/or protein levels of these subunits of the p400/Tip60 complex. To further determine whether *p21* is the gene responsible for growth deficiency and G1/S arrest in BRD8-depleted cells, we depleted BRD8 in p21−/− HCT116 cells and assayed cell viability. Viability of HCT116 p21−/− cells decreased significantly following the knockdown of BRD8 by BRD8-targeting siRNA compared to control siRNA (Fig. 5a). Cell cycle analysis showed that BRD8 knockdown significantly increased cell populations in the G2-phase (G2-phase: 24.06% in control cells to 35.49% in BRD8 depleted cells), and also induced a significant decrease in the cell population with S-phase DNA content (S-phase: 51.58% in control to 36.35% in BRD8 depleted cells) (Fig. 5c). We further examined the effect of BRD8 knockdown on the induction of apoptosis in HCT116 p21−/− using Annexin V-FITC/propidium iodide (PI) staining followed by FACS analysis. HCT116 p21−/− cells showed sensitivity to the transfection process itself as control siRNA transfection resulted in some cell death induction (Fig. 5b). Nevertheless, knockdown of BRD8 caused an increase in the population of both Annexin V positive/PI negative early apoptotic and Annexin V positive/PI positive late apoptotic and dead cells (Fig. 5b). The effect of BRD8 knockdown on p21−/− cells suggests that the up-regulation of *p21* is partially responsible for the G1/S phase arrest in BRD8-depleted p53+/+ cells with intact *p21*, but besides the up-regulation of *p21*, other molecular mechanisms exist to explain the proliferation defects, and cell death observed in these cells.

### BRD8 is required to prevent DNA damage in non-stressed cells

The above experiments indicate that the knockdown of BRD8 induces p53-dependent apoptosis in non-stressed cells. This begs the question as to what triggers programmed cell death in these cells. It has been shown that apoptosis can be triggered in response to DNA damage^40^. In order to investigate this possibility, we first assayed whether BRD8 knockdown affects p53 accumulation. As mentioned above, analysis of *p53* expression indicated that both mRNA and protein levels of *p53* was elevated in BRD8 depleted cells (Fig. 4b,e). p53-*Ser*15 phosphorylation is one of the canonical modifications in response to DNA damage that can be mediated by the activation of the master PI3K-like kinases of the DDR including ATM^41^. In response to DNA damage, activation of ATM itself occurs through phosphorylation of residue *Ser*1981^41^. As shown in Fig. 4e, BRD8 knockdown resulted in an increase in p53-*Ser*15 phosphorylation, ATM protein level and ATM-*Ser*1981 phosphorylation in p53+/+ cells. Immunoblot analysis also confirms that the ATM-branch of the DDR is activated in BRD8 depleted p53−/− cells, as ATM is phosphorylated at *Ser*1981 (Fig. 4f).

**Figure 6:**
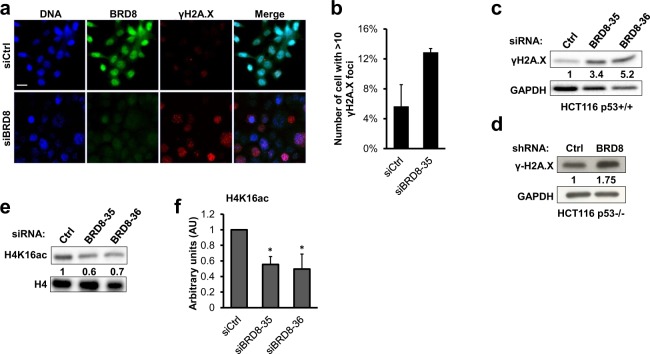
BRD8 knockdown causes spontaneous DNA damage and decreases chromatin- bound H4K16 acetylation. (**a**) Detection of DNA damage foci in BRD8 knockdown cells. HCT116 p53+/+ cells were transfected with BRD8-targeting (siBRD8-35) or control (Ctrl) siRNAs. Cells were fixed and γH2A.X foci (red) and BRD8 (green) localization were monitored by immunostaining. Nuclei were counterstained with DAPI. Scale bar is 20 μm. (**b**) The histogram represents the percentage of cells with more than 10 γH2A.X foci. Total cell extracts were subjected to immunoblot assays using the indicated antibodies 48 h following knockdown with BRD8-targeting (siBRD8-35 and siBRD8-36) or control (Ctrl) siRNAs in HCT116 p53+/+ cells (**c**) and with BRD8-targeting (shBRD8) or control (Ctrl) shRNAs in HCT116 p53−/− cells (**d**). (**e**) Total histone extracts of HCT116 p53+/+ were subjected to immunoblot assays using indicated antibodies 48 h post transfection with BRD8-targeting (siBRD8-35 and siBRD8-36) or control (Ctrl) siRNAs. (**f**) Densitometric analysis of Western blot with average intensity values from three independent experiments. Intensities were calculated using Image Lab and normalized to the intensities of total histone H4. The mean ± SD from three independent experiments are shown. An ANOVA test followed up with a Dunnett analysis was used to compare each mean to its relative control. *P ≤ 0.05; compared to control.

Moreover, DNA damage, particularly DSBs, induces the phosphorylation of histone H2A.X on *Ser*139 (γ-H2A.X)^42^ that can be mediated by activated ATM^43^. To investigate whether spontaneous DNA damage occurs in BRD8-depleted cells, we monitored γ-H2A.X foci formation by immunofluorescence staining and immunoblotting. Similar to previously published data^44^, the BRD8 antibody stained the chromatin in nuclei and was excluded from nucleoli (Fig. 6a upper panel). In BRD8-depleted cells, the fluorescence intensity of BRD8 antibody decreased markedly indicating the efficiency of our knockdown (Fig. 6a lower panel). When we monitored γ-H2A.X foci formation, the fluorescence intensity of γ-H2A.X was clearly higher in siBRD8 than in control cells (Fig. 6a lower panel). We further analyzed γ-H2A.X foci formation by manual counting in acquired images, and cells containing more than 10 foci were scored as positive. The percentage of cells with more than 10 γ-H2A.X foci was higher following knockdown of BRD8 (Fig. 6b). In addition, immunoblot analysis of γ-H2A.X also confirms that the levels of γ-H2A.X increased in both p53+/+ (Fig. 6c) and p53−/− cells (Fig. 6d). These data suggest that BRD8 is required to prevent DNA damage in non-stressed cells.

As the p400/Tip60 complex is shown to be recruited to DNA damage sites and plays a direct role in the protection of cells against DNA damage^21,22^, we then asked whether BRD8 itself is recruited to DNA damage sites on chromatin. To examine this possibility, HCT116 cells were treated with 1 μM camptothecin (CPT), an inhibitor of topoisomerase I, which induces DSBs at collapsed replication forks. Despite the strong induction of γ-H2A.X foci, no BRD8 accumulation at DNA damage sites marked with γ-H2A.X were detected (Fig. S3a enlarged insets). To further validate these results, we used UV-laser microirradiation (laser striping) to create localized tracks of DNA damage in HCT116 and U2OS cells to better study BRD8 localization in response to DNA damage^45^. BRD8 did not accumulate at DNA damage sites and no change in the pattern of BRD8 localization was detected at the indicated time points (Fig. S3b). This is in agreement with previously published data that categorized BRD8 amongst the BRD proteins that do not accumulate at DNA lesions^44^.

Even though we were not able to detect BRD8 accumulation at DNA lesions, previous studies found other subunits of p400/Tip60 complex on chromatin adjacent to DSBs^16,21,24,46–48^. We sought to further explore the potential role of BRD8 in the recruitment of p400/Tip60 complex on chromatin by examining the acetylation of lysine 16 at the N-terminal tail of histone H4 (H4K16), as Tip60 is responsible for this modification at DNA damage sites^49^. As shown in Fig. 6e and f knockdown of BRD8 led to a reproducible decrease of chromatin-bound H4K16ac compared to control, whereas there was no difference in Tip60 transcription (Fig. S2c). Our results suggest that BRD8 may play a role in the recruitment or stabilization of p400/Tip60 complex on chromatin thereby facilitating the acetylation of H4K16.

### BRD8 knockdown causes a severe decrease in CHK1 protein levels

**Figure 7:**
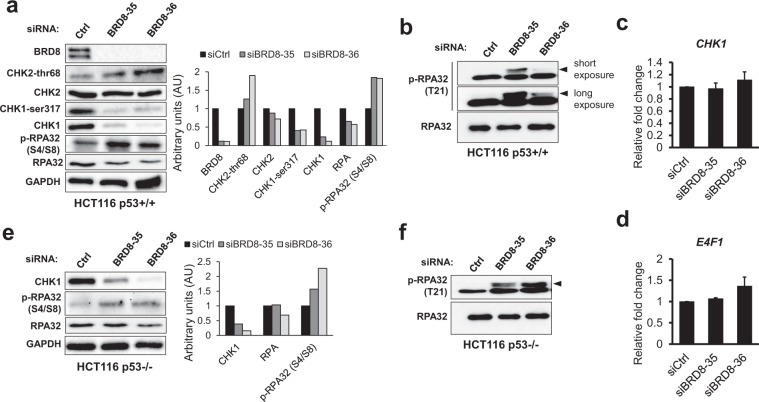
BRD8 knockdown activates CHK2 but abrogates CHK1. Total cell extracts of HCT116 p53+/+ (**a** and **b**) and p53−/− (**e** and **f**) were subjected to immunoblot assays using indicated antibodies 48 h post-transfection with BRD8-targeting (siBRD8-35 and siBRD8-36) or control (Ctrl) siRNAs. The graphs represent densitometric analyses normalized to *GAPDH*. (**c** and **d**) RT-qPCR assay showing mRNA expression levels of *CHK1* and *E4F1*. The mean ± SD from three independent experiments are shown. The arrows indicate hyper-phosphorylated RPA32 species containing pT21.

In response to DNA damage, ATM and ATR (ATM and Rad3-related), which belong to the PI3K-like kinase family of proximal transducer kinases, activate checkpoints to arrest cell cycle progression until DNA integrity is restored. We sought to investigate the status of the ATM- and ATR-dependent branches of the DDR in BRD8-depleted cells by monitoring the phosphorylation level of their canonical substrates CHK2 and CHK1 respectively. Cells were transfected with siBRD8 and immunoblots for CHK2 phosphorylated on *Thr* residue 68 and CHK1 phosphorylated on *Ser* 317 were performed. In agreement with ATM being activated in BRD8-depleted cells, phospho *Thr* 68 CHK2 levels were increased in BRD8 knockdown cells. In sharp contrast, the level of phospho *Ser* 317- CHK1 dropped in BRD8-depleted cells (Fig. 7a). We next monitored the levels of the CHK2 and CHK1 kinases themselves. While total CHK2 was unaffected by BRD8 depletion, CHK1 levels strongly decreased in BRD8 knockdown cells (Fig. 7a). To determine whether BRD8 is involved in the transcriptional regulation of CHK1 we assayed the transcription levels of *CHK1*. As shown in Fig. 7c, mRNA levels of *CHK1* were not affected in BRD8 knockdown cells. *CHK1* transcription can be regulated by the transcription factor E4F1^50^. E4F1 also interacts with and protects the checkpoint kinase 1 (CHK1) protein from degradation^51^. Hence, we monitored mRNA level of *E4F1* in BRD8 knockdown cells. As shown in Fig. 7d, mRNA levels of *E4F1* were not affected by cellular depletion of BRD8.

Because CHK1 downregulation or inhibition strongly induces DNA replication stress^52^, we investigated the levels of RPA phosphorylation, an established marker of replication impairment in BRD8-depleted cells^52–54^. In response to replication stress, the N-terminus of RPA32 is sequentially hyper-phosphorylated on at least 9 sites by the PIKK family protein kinases ATM, ATR and DNA-PK. The regulation of these phosphorylation events is complex and different kinases may modify the same residues. Phosphorylation on *Ser* 4/8 is typically carried out by DNA-PK whereas *Thr* 21 can be targeted by ATM, ATR and DNA-PK. Phosphorylation of RPA32 on these sites is a well-established marker of replication stress-associated DSBs^55^. As shown in Fig. 7a,b replication stress is detectable as enhanced RPA32 hyper-phosphorylation on residues S4/8 and T21 in BRD8-KD HCT116 p53+/+ cells. The decrease in CHK1 abundance and phosphorylated RPA32 was also present in BRD8-depleted HCT116 p53−/− cells indicating that this phenomenon is not related to p53 status (Fig. 7e,f).

## Discussion

In this study, we describe a mechanism that underlies a previously reported cell proliferation deficiency observed in BRD8-depleted cells^11^. Importantly, we have shown that cellular depletion of BRD8 leads to the accumulation of spontaneous DNA damage, which triggers the DDR under normal growth conditions. We demonstrate that p53 is activated with concomitant target gene transcription in BRD8-depleted cells and this accounts for upregulation of *p21* and the *Puma*, *p53DINP1*, and *Fas* pro-apoptotic genes. These events lead to a G1/S cell cycle arrest and apoptosis in a p53-dependent manner. Accordingly, in the absence of either *p53* or *p21*, cells that were depleted for BRD8 showed G2/M-phase arrest as well as growth deficiency. Our results strongly suggest that loss of BRD8 impairs the cell’s ability to repair DNA, thereby activating the G1/S checkpoint in p53+/+ cells, and the G2/M checkpoint in the absence of either p53 or p21.

The p400/Tip60 chromatin remodeler/HAT complex and histone H2A.Z have both been implicated in the regulation of p53 target gene expression and in DNA damage-induced apoptosis^37,56–58^. Previous studies in our laboratory have demonstrated that p400-dependent deposition of H2A.Z at the distal p53-binding site of the *p21* promoter inhibits p53- dependent *p21* transcription and thereby inhibits replicative senescence in primary human fibroblasts, but not in cells with deficient p53^37^. Moreover, knockdown of H2A.Z as well as p400 results in an increase in *p21* expression and cellular senescence through the p53/p21 pathway^37,38^. In addition, loss of MRG15 was shown to limit neural stem/progenitor cell proliferation via increased expression of *p21*^39^. It is well established that cells lacking either H2A.Z or components of p400/Tip60 complex are hypersensitive to DNA damage and show defects in DNA damage repair^16,22–24,59,60^. Our study shows that although BRD8 is a *bona fide* component of p400/Tip60, it may exhibit a novel and specific role in the maintenance of genome stability, which promotes unimpeded cell cycling and avoids p53-dependent apoptosis. It is important to note that our data eliminates the possibility that BRD8 negatively controls p53-target gene expression by modulating the expression of H2A.Z or the p400, Tip60 and MRG15 subunits of the p400/Tip60 complex. Even though there is a clear involvement of BRD8 in genome stability, we were not able to detect BRD8 accumulation at DNA damage sites induced by camptothecin or UV-laser microirradiation. Although this is in agreement with previously published data^44^, several other subunits of the p400/Tip60 complex including Trrap^16,61^, p400^21^, Tip60^16^, DMAP1^48^, Ruvbl1/2^47^, MRG15^46^, and ANP32E^24^ were shown to be recruited to DSBs. However and importantly, we observed a significant reduction in global H4K16 acetylation in BRD8-depleted cells, possibly indicating that BRD8 could be involved in the recruitment of Tip60 at those chromatin sites. Optimal H4K16 acetylation would be necessary for the remodeling of chromatin during DNA repair events. Accordingly, cellular depletion of MRG15 was also reported to globally reduce chromatin-bound Tip60 and decrease H4K16 acetylation^62^. Repair of damaged DNA requires remodeling of local chromatin structure to facilitate the access of the repair machinery to sites of DNA damage and H4K16ac is particularly important for decondensation of chromatin^63^.

Finally, and consistently with our observations in human cells, it has been reported that yeast cells mutated for the BRD8 homologous genes, *bdf1* and *bdf2*, display DNA damage accumulation, significant reduction in H4 acetylation levels, and deficiency in recovery of replication fork breakage during S-phase^64^. These results suggest that Bdf1/2 modulate chromatin structure to coordinate DNA replication and S-phase stress response through proper acetylation of H4 and deposition of H2A.Z. Thus, our results suggest a similar role for BRD8 in human cells to prevent replicative stress-induced DNA damage.

Notably, in BRD8 depleted cells, *CHK1* and *E4F1* mRNA levels remain unchanged but CHK1 protein levels are severely decreased. CHK1 functions as a major effector of S-phase checkpoint in response to replication and genotoxic stresses. However, once CHK1 is activated via phosphorylation by ATR, phosphorylated CHK1 undergoes degradation through the proteasome^65^ and/or chaperone-mediated autophagy^66^. Moreover, a CHK1 protein level decrease or its inactivation could lead to replicative stress, which would then cause DSBs and activate the DDR^67^. Whether CHK1 is degraded in response to genotoxic and/or replication stress in BRD8-depleted cells or whether BRD8 itself plays a more direct role in the post-transcriptional regulation of CHK1 levels remains to be determined. Either of these scenarios would lead to the enhanced replication stress and DNA damage that we observed in the absence of BRD8.

Taken together our observations indicate that BRD8 may be required for DNA repair and/or for preventing DNA damage. This most probably occurs through the recruitment and stabilization of the p400/Tip60 complex within chromatin and also likely via the regulation of CHK1 protein levels to ensure DNA replication. Enhanced replicative stress owing to CHK1 deficiency, combined with an inability to efficiently remodel chromatin at sites of damage would result in a gradual accumulation of DNA damage that would ultimately trigger cell-cycle arrest and apoptosis. Further experiments are required to clarify the role of BRD8 in the checkpoints and genome stability.

## Materials and Methods

### Cell Culture

HCT116 40.16 (p53+/+), HCT116 397.2 (p53−/−) and HCT116 (p21−/−) cell lines were a generous gift from Dr. Bert Vogelstein (Howard Hughes Medical Institute at the Hopkins-Kimmel Comprehensive Cancer Center, Baltimore 21287, USA). U2OS were obtained from the American Type Culture Collection (ATCC). All the cell lines were maintained in Dulbecco’s modified Eagle’s medium (Wisent) supplemented with 10% fetal bovine serum (Sigma), 0.2 u/ml penicillin G and 100 mg/ml streptomycin (Invitrogen). All cell cultures were incubated at 37 °C in a humidified incubator containing 5% CO_2_.

### BRD8 knockdown

#### siRNA

siRNA for BRD8 was purchased from Sigma (SASI_Hs01_00131635 and SASI_Hs01_00131636; target sequences start at nucleotides 2223 and 1473, respectively). Non targeting control siRNA was purchased from Qiagen. Transfection was performed with lipofectamine RNAiMAX transfection reagent (Life Technologies) following manufacturer’s instructions. Briefly, for transfection of one well in a 6-well plate, 1.25 μl of 20 μM siRNA and 5 μl of lipofectamine RNAiMAX were mixed in 500 μl Opti-MEM reduced serum medium (Gibco by Life Technologies) and added to the wells. Then 200,000 cells in 2 ml antibiotics-free DMEM medium were added to the siRNA complexes.

#### shRNA

To knockdown BRD8, a mix of two shRNAs directed against BRD8 (TRCN0000229929 + TRCN0000229926, Sigma) and a control shRNA in Plko.1-based lentiviral vector were used. Cells were infected immediately following cell passage with either the mix of two lentiviruses containing BRD8 shRNAs or control shRNA in the presence of polybrene (8 µg/ml) for 24 h. After 48 hours, cells were treated or not with 250 μM daunorubicin (Sigma). 56 hours following infections, the cells were collected for subsequent experiments.

### Immunoblotting

Briefly, cells were washed once with PBS, harvested and re-suspended in lysis buffer containing 50 mM Tris-HCl pH 7.5, 150 mM NaCl, 1% triton X-100, 0.5% Na-deoxycholate, 0.2% SDS, supplemented with 1 mM PMSF, 1X Roche Complete protease inhibitor cocktail, and phosphatase inhibitor cocktail (ThermoFisher Scientific) and passed 5 times through a 23G1 needle. Lysis was performed by incubation at 4 °C for 1 hour on a rotator. Cell lysates were centrifuged at 14,000 rpm for 10 minutes at 4 °C and the supernatants were dosed using the Bradford method and then boiled at 95 °C with SDS loading buffer for 10 minutes.

Western blots of H2A.Z and H3 were performed on histone extracts. Cells were washed with PBS, collected, resuspended in Triton extraction buffer (0.5% Triton X-100, 2.5 mM PMSF, 0.02% NaN_3_, 1X Roche Complete protease inhibitor cocktail), incubated on ice for 10 min, and centrifuged at 7,500 rpm for 8 min at 4 °C. The pellet was washed with 500 µl of Triton extraction buffer and then centrifuged at 7,500 rpm for 8 min at 4 °C. The pellet was resuspended in 0.2 N HCl (50 µl for 4 million cells) and incubated overnight at 4 °C. The next day, histone extracts were cleared by centrifugation at 7,500 rpm for 8 min at 4 °C. The supernatant was collected and dosed.

Densitometry analysis were performed using Image Lab (Bio-Rad) and/or ImageJ softwires. Intensities were normalized to the intensities of their corresponding loading controls and relative fold changes were calculated on siCtrl lane.

All the antibodies used are listed in Table S1.

### Isolation of RNA and quantitative PCR

Total RNA was extracted using the Quick-RNA™ MicroPrep kit (Zymo Research) according to the manufacturer’s protocols. One microgram of RNA was reverse transcribed using M-MuLV reverse transcriptase (Enzymatics) and random hexamers (Sigma) according to the manufacturers’ protocols. Samples were then subjected to quantitative PCR (qPCR) using CFX Connect Real Time System (Bio-Rad Laboratories). The relative abundance of target mRNA was calculated according to the ∆∆ cycle threshold method (∆∆Ct). mRNA expression levels of the housekeeping gene *36B4* gene (also called ribosomal phosphoprotein P0 (RPLP0)) were used as an internal control to normalize each qPCR reaction. The relative expression levels were calculated as fold enrichment of treated cells over the control cells. Experiments were performed as independent biological triplicates and data are presented as mean ± SD. The complete list of primers used in RT-qPCR experiments is provided in Table S2.

### Cell viability assay

Crystal violet staining was performed to assess cell viability. Cells were incubated in 48-well plates at 2.0 × 10^4^ cells per well and cell viability was measured at the indicated time points. For crystal violet staining, the culture medium was removed; the cells were washed with PBS and fixed with 4% formaldehyde in PBS at room temperature for 10 min, washed with PBS and stained with 0.1% crystal violet for 30 min at room temperature. The cells were then washed with water, after which the water was removed and the cells were dried out. Crystal violet dye was dissolved in 200 μl of 10% acid acetic and transferred to 96-well plate. Optical densities were measured at a wavelength of 590 nm using a Bio-tek µQuant Monochromatic Microplate Spectrophotometer. Experiments were performed as independent biological triplicates in technical duplicates and data are presented as mean ± SD.

### Cell cycle analysis

Fluorescence-activated cell sorting (FACS) analysis was used for cell cycle profiling. FACS samples were harvested with trypsinization, washed twice with PBS, fixed with −20 °C 70% ethanol and stored at 4 °C until use. The cells were rehydrated with PBS, treated with ribonuclease A (10 μg/ml) and propidium iodide (50 μg/ml) in PBS and subjected to FACS analysis. FACS analysis was performed using FACS-calibur flowcytometer (BD Biosciences, Mississauga, Ontario, Canada). Analysis of data was performed with FCS Express 5 software.

### Apoptosis detection

Apoptosis analyses were performed using the Annexin V-FITC Apoptosis Detection Kit (Sigma) according to manufacturer’s instructions. Briefly, cells were harvested by trypsinization, washed twice with PBS, re-suspended at a concentration of 1 × 10^6^ per milliliter in 1 ml 1X binding buffer, then 10 μl propidium iodide and 5 μl FITC conjugated AnnexinV were added, incubated for 10 minutes at room temperature in the dark and subjected to FACS analysis. FACS analysis was performed using FACS-calibur flowcytometer (BD Biosciences, Mississauga, Ontario, Canada). Analysis of data was performed with BD CellQuest software.

### Laser microirradiation

For laser microirradiation, cells were grown directly on culture slides (Millipore). Cells were pre-sensitized with 10 μM BrdU for 24 hours at 37 °C. Before microirradiation, media was replaced with DMEM without phenol red. Laser microirradiation was performed using MMI Cellcut Plus 355 nm Laser Capture microscope (Molecular Machines & Industries). Laser power was 50–55% of maximum to generate DNA damage restricted to the laser paths. Following damage, cells were incubated for the indicated times for recovery and processed for immunofluorescence staining and analysis.

### Immunofluorescence

For immunostaining, cells were grown on coverslips or directly on culture slides (for Laser microirradiation). Cells were pre-extracted with 0.25–0.125% Triton X-100 in PBS on ice, and then fixed with 3% paraformaldehyde in 2% sucrose solution for 15 minutes at room temperature. Then cells were permeabilized with ice cold 0.5% Triton X-100 in PBS on ice for 5 minutes, blocked in 3% BSA and 0.05% Tween-20 in PBS prior to incubation with primary antibodies. After 3 washes with 0.05% Tween-20 in PBS, cells were incubated with secondary antibodies. For nuclei staining, 1 µg/mL DAPI solution was used. Coverslips mounted onto microscope slides using ProLong® Diamond Antifade Mountant (Life Technologies). All the antibodies used are listed in Table S1.

### Statistical analysis

The mean and standard deviations from at least three independent experiments were calculated and two-sample t-Tests assuming unequal variances or One-way ANOVA test followed up by a Dunnett’s post hoc analysis used to compare the mean between groups. Results are presented as the mean ± SD; p values ≤ 0.05 were considered statistically significant.

## Electronic supplementary material

Supplementary material
