# Supplementary material for: Cellular Depletion of BRD8 Causes p53-Dependent Apoptosis and Induces a DNA Damage Response in Non-Stressed Cells
Source: Sci Rep. 2018 Sep 20;8:14089. doi: 10.1038/s41598-018-32323-3 (PMC6147888; doi:10.1038/s41598-018-32323-3)
Supplement: Supplementary file 1 — Supplementary material [file 41598_2018_32323_MOESM1_ESM.docx]

**SUPPLEMENTARY DATA**

**Cellular Depletion of BRD8 Causes p53-Dependent Apoptosis and Induces a DNA Damage Response in Non-stressed Cells**

Anahita Lashgari, Myriam Fauteux, Alexandre Maréchal, and Luc Gaudreau*

Département de biologie, Université de Sherbrooke, 2500 Boulevard de l’Université, Sherbrooke, QC, J1K 2R1, Canada

***Corresponding author: Luc.Gaudreau@USherbrooke.ca**

This PDF file includes:

Supplementary Figures Legends

Figure S1 to S3

Table S1 and S2

**Supplementary Figure Legends**

**Figure S1 BRD8 Knockdown using shRNA induces p53-dependent apoptosis in HCT116 cells.** BRD8 was depleted in HCT116 cells with BRD8-targeting or control (Ctrl) shRNA. Apoptosis in HCT116 p53+/+ (a) and HCT116 p53-/- (b) cells were quantified by FACS analysis of Annexin V and PI double staining 72h post transfection with shRNA targeting BRD8 or control shRNA (Ctrl). mRNA expression levels of *BRD8* in HCT116 p53+/+ (c) and p53-/- (d) cells before and after knockdown. Immunoblot showing the BRD8 protein levels and cleaved PARP in HCT116 p53+/+ (e) and HCT116 p53-/- (f) cells in BRD8 depleted cells. Data are the mean ± SD from three independent experiments. Two-sample t-Test assuming unequal variances was used for statistical analysis. ***** P≤ 0.05; ** P ≤0.005; ns = non-significant compared to control.

**Figure S2 The protein levels of H2A.Z and transcription levels of p400, Tip60 and MRG15 are not significantly affected in BRD8-depleted cells.** HCT116 cells were transfected with two siRNAs targeting BRD8 (siBRD8-35 and siBRD8-36) or control (Ctrl) siRNA for 48h. mRNA expression levels of p400 (a), Tip60 (b), MRG15(c) and H2A.Z (d) measured by RT-qPCR . The mean ± SD from three independent experiments are shown. (e) Histone extracts of HCT116 p53+/+ were subjected to immunoblot assays using indicated antibodies. ANOVA follow up with Dunnett’s test was used to compare each mean to control. * P≤0.05; ns = non-significant compared to relative control.

**Figure S3 Immunofluorescence analysis of BRD8 localization in response to DNA damage.** (a) HCT116 p53+/+ cells were treated with camptothecin (1μM) for 1 hour, pre-extracted, fixed and γH2A.X (red) and BRD8 (green) localization were monitored by immunofluorescent staining. Enlarged insets are in the right column. Scale bar is 20μm. (b) UV laser microirradiation was performed using a 355 nm laser to create DNA damage in HCT116 and U2OS cells presensitized with BrdU. Cells were pre-extracted and fixed at indicated times. γH2A.X (red) and BRD8 (green) were detected by immunofluorescent staining. Nuclei were counterstained with DAPI. Scale bar is 5μm.

**Tables**

**Table S1 Antibodies**

|  | **Company** | **Reference** |  |
| --- | --- | --- | --- |
| **Actin** | Sigma | A2066 | WB |
| **ATM** | Bethyl-rabbit | A300-299A | WB |
| **ATM-Ser 1981** | Epitomic | 2152-1 | WB |
| **BRD8** | Abcam | ab17969 | WB/IF |
| **Chk1** | Cell Signaling | 2345 | WB |
| **Chk1-Ser317** | Cell Signaling | 2344 | WB |
| **Chk2** | Millipore | 05-649 | WB |
| **Chk2-Thr68** | Cell Signaling | 2661 | WB |
| **GAPDH** | Ambion | AM4300 | WB |
| **H2A.X-Ser139** | Santa Cruz | sc-9718 | WB |
| **H2A.X-Ser139** | Millipore | 05-636 | IF |
| **H2A.Z** | Abcam | ab4174 | WB |
| **H3** | Abcam | ab1791 | WB/ChIP |
| **H4** | Abcam | ab177840 | WB |
| **H4 acetyl K16** | Abcam | ab109463 | WB |
| **p400** | Abcam | ab5201 | WB |
| **p53** | Santa Cruz | sc-6243 | WB |
| **P53-ser15** | Cell Signaling | 9284S | WB |
| **PARP** | Santa Cruz | sc-74470 | WB |
| **pRPA32 S4/8** | Bethyl | A300-245A | WB |
| **pRPA32 T21** | Epitomics | 3237-1 | WB |
| **RPA32** | Santa Cruz | Sc-56770 | WB |

**Table S2 RT-qPCR Primers**

|  | **Forward** | **Reverse** |
| --- | --- | --- |
| ***36B4*** | CGACCTGGAAGTCCAACTAC | ATCTGCTGCATCTGCTTG |
| ***BAX*** | GTTGTCGCCCTTTTCTACTTT | GGAGGAAGTCCAATGTCCAG |
| ***BRD8*** | ATGGTGGGGAGATACAGCAA | AGTATGTGGATCCCCCACAG |
| ***CDKN1A (p21)*** | GGAGACTCTCAGGGTCGAAA | GGATTAGGGCTTCCTCTTGG |
| ***CDKN1B (p27)*** | ACCAGAGGCAGTAACCATGC | CCTGTAGGACCTTCGGTGAC |
| ***CHK1*** | CCCGCACAGGTCTTTCCTT | GGCTGGGAAAAGCTGATCC |
| ***E4F1*** | TCTCTGGCAGCAGACATCAG | GCCACGATGACCTCTTTGAT |
| ***Fas*** | GGTTGGTGGACCCGCTCAGTACGGAG | CTGGTTCATCCCCATTGACTGTGCAGTCC |
| ***H2A.Z*** | TGGGAAGAAAGGACAACAGAA | TGGAATCACCAACACTGGAC |
| ***GAPDH*** | GGCCTCCAAGGAGTAAGACC | AGGGGAGATTCAGTGTGGTG |
| ***MDM2*** | CAAGTTACTGTGTATCAGGCAGGG | TCTGTTGCAATGTGATGGAAGG |
| ***MRG15*** | CACCCATGTCCCAGGTGTAT | GCAAGGCTCTTCTCATCCAG |
| ***p400*** | AACTTTTGCCAAACCCACAG | GTGAGGTTCAAAGGCAGCTC |
| ***p53*** | TCATTCAGCTCTCGGAACATC | CTCACCATCATCACACTGGAA |
| ***p53DINP1*** | TTCCTGTTTACCGGCATCTC | AGCTCTTGGGTTGTTCCAGA |
| ***PUMA*** | GACCTCAACGCACAGTACGAG | AGGAGTCCCATGATGAGATTG |
| ***TIGAR*** | CAAAGCGCTAAGTGAGCTGAG | AAAAAGTCTATTCCACGCATTTTC |
| ***Tip60*** | CATCCTCCAGGCAATGAGAT | ACTTGGCCAAAAGACACAGG |

**Figure S1**


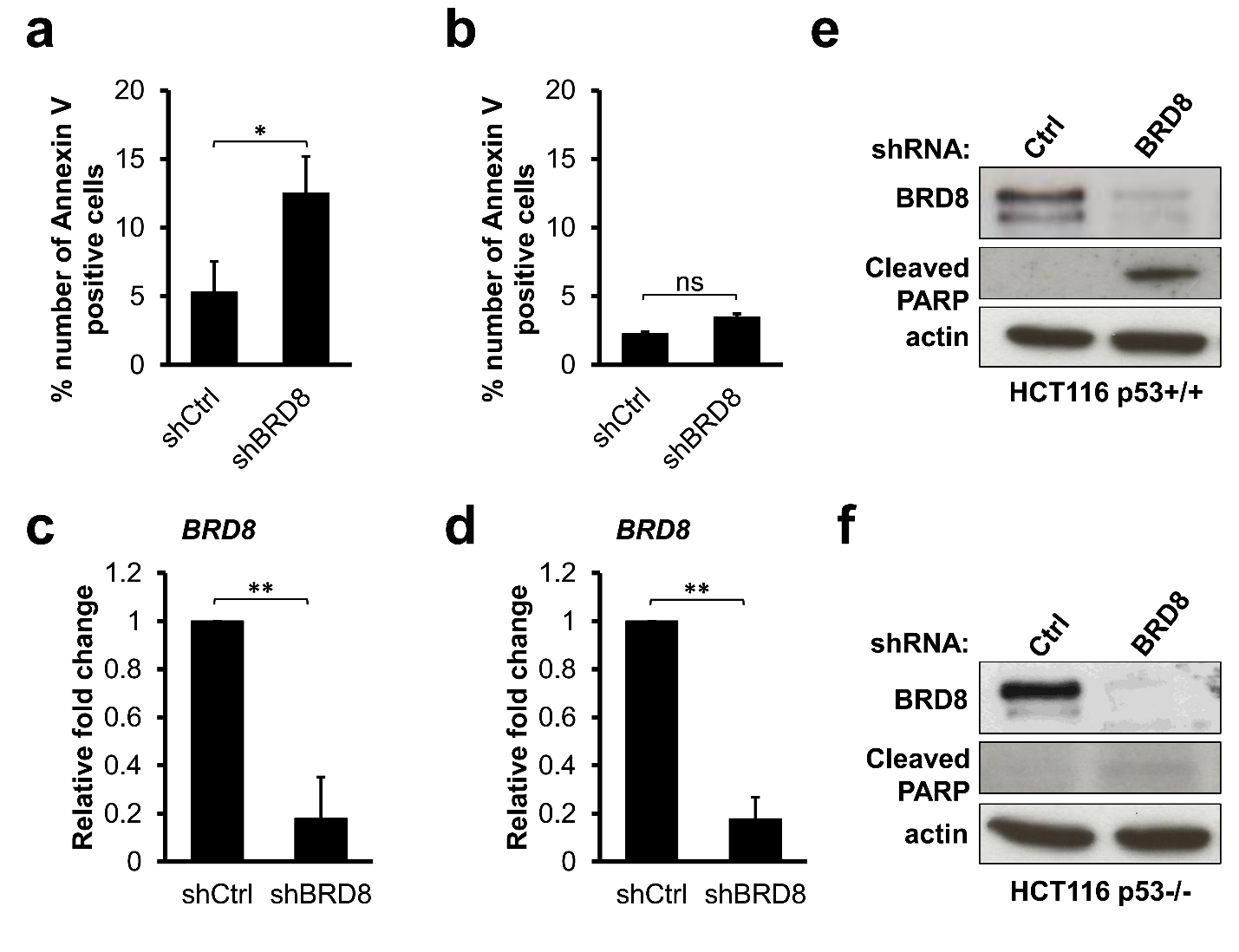


**Figure S2
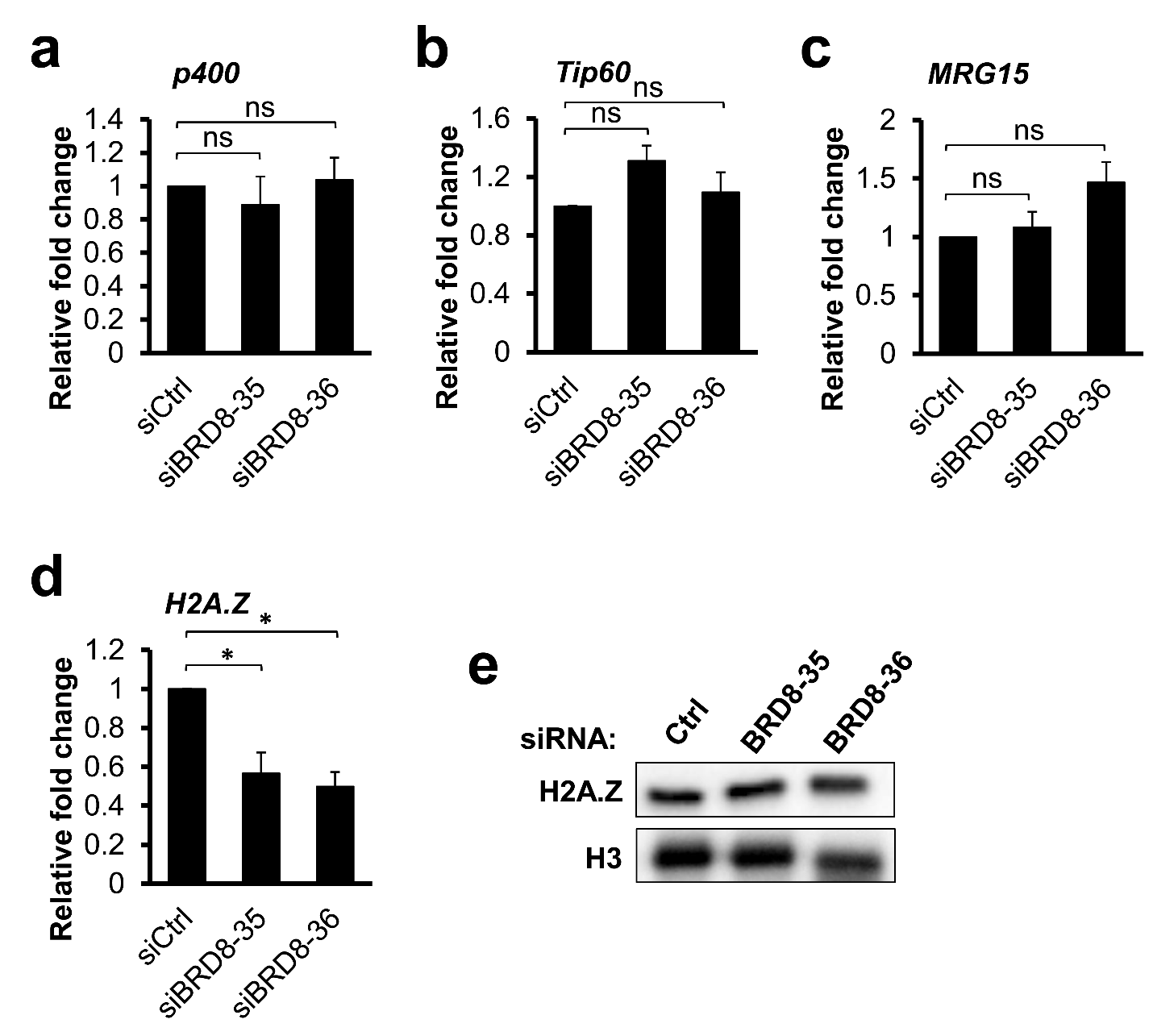
**

**Figure S3**

**
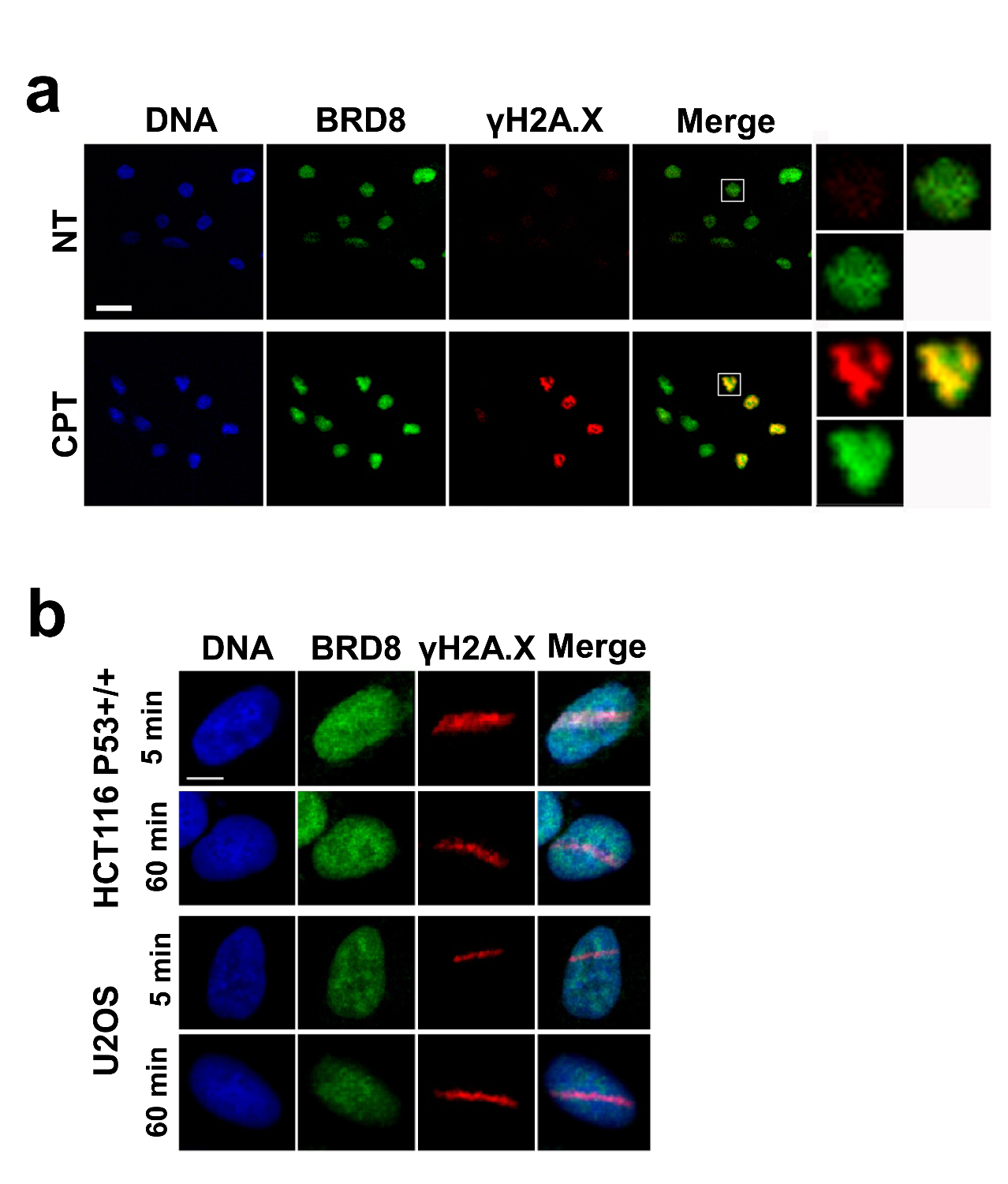
**
